# Social comparison contributes to work exhaustion in the context of workplace AI use: A three-wave follow-up study of Finnish workers

**DOI:** 10.1016/j.ssmph.2026.101945

**Published:** 2026-06-29

**Authors:** Iina Savolainen, Teijo Osma, Roope Grönroos, Moona Heiskari, Atte Oksanen

**Affiliations:** Faculty of Social Sciences, Tampere University, Kalevantie 4, 33100, Tampere, Finland

**Keywords:** Artificial intelligence, Work exhaustion, Social comparison, Longitudinal

## Abstract

**Background:**

The rise of artificial intelligence (AI) is transforming work tasks and social relations within organizations. Work exhaustion is increasing, and AI may heighten employees' sense of adaptability and social comparison. This three-wave longitudinal study examines employees’ work exhaustion as an outcome of AI use at work, perceived AI readiness, and social comparison tendencies.

**Methods:**

Data were drawn from a national survey of employed adults aged 18–65 years in Finland. Baseline data were collected in fall 2024 (N = 2109; 50% men; mean age 42·6 years), with biannual follow-ups. Work exhaustion (Maslach Burnout Inventory), social comparison orientation (INCOM), perceived AI readiness, and AI use at work were assessed. Within-between and multilevel mixed effects regression models were used to estimate within- and between-person associations over time.

**Findings:**

Frequent AI use at work was not associated with work exhaustion, whereas social comparison tendency showed robust associations with greater exhaustion at both the within- (β = 0·02, [95% CI 0·01 to 0·04], P = 0·001) and between-person (B = 0·20, [95% CI 0·15 to 0·25], P < 0·001) levels. Workers with higher perceived AI readiness reported lower exhaustion between individuals in the first model (B = −1·41, [95% CI −2·17 to −0·64], P <0·001) and within individuals (B = −0·02, [95% CI -0·04 to −0·01], P = 0·002) and between individuals in the second model (B = −0·16, [95% CI −0·21 to −0·11], P <0.001). No interaction effects were observed between AI use at work and AI readiness or social comparison in the primary models. However, exploratory analyses suggested that among employees with high social comparison tendencies, frequent AI use at work was associated with elevated exhaustion (B = 0·05, [95% CI 0·00 to 0·09], P = 0·035).

**Interpretation:**

Findings suggest that individual psychosocial dispositions may play an important role in experiences of exhaustion in AI-augmented workplaces. At the same time, perceived AI readiness may function as a personal resource associated with lower exhaustion.

## Highlights

• Three-wave longitudinal study of Finnish workers' exhaustion amid AI disruption.
• Frequent AI use at work did not predict work exhaustion.
• Higher general AI readiness was associated with lower work exhaustion.
• Social comparison orientation was linked to higher work exhaustion.
• AI use predicted exhaustion among workers high in social comparison tendencies.

## 1 Introduction

Work exhaustion is rising among professionals across sectors (Fernández-Suárez et al., 2021; National Academies of Sciences, Engineering, and Medicine; Policy and Global Affairs; Committee on Women in Science, Engineering, and Medicine, 2025). According to the job demands-resources model (Demerouti et al., 2001), work exhaustion is a core component of burnout and arises when job demands such as physical, affective, and cognitive demands, including work overload, exceed employees’ capacity to cope, given available job resources such as job security and control. The consequences of work exhaustion are wide-ranging, manifesting in reduced work performance, increased mental-health burden, impaired functioning, workplace absence, and disability (Petrou et al., 2015; Salvagioni et al., 2017).

The COVID-19 pandemic substantially contributed to increased burnout among workers since 2020 (Galanis et al., 2021; Trinidad, 2025), but more recent developments in working life have been attributed to digital transformation (Bankins, Ocampo, et al., 2024). In particular, the rapid growth and integration of artificial intelligence (AI) into work processes has introduced new psychosocial demands and concerns that may influence employee well-being (Savolainen et al., 2026; Triantoro, 2025). Much of the recent literature has examined how digital technologies and AI contribute to stress and techno-overload (Kumar, 2024; Issa et al., 2024). However, less is known about the underlying social mechanisms through which these technologies and AI may also contribute to work exhaustion.

As AI tools become increasingly embedded in daily work tasks, employees must adapt to new technologies while navigating competitive environments in which digital competence, particularly AI-related skills, influences perceptions of performance, career advancement, or job security (Dumlao Jr et al., 2024; Lábaj et al., 2025). Although workers are unlikely to lose their jobs directly to AI, concerns about being replaced or outperformed by colleagues who are more proficient in using AI tools and thus remain employable, may persist (Balcerzak et al., 2025; Jaiswal et al., 2022). Relying on the JDR framework, we conceptualize AI readiness as a personal resource that reflects employees' perceived competence in understanding and using AI technologies in their work. AI readiness is related to constructs such as general self-efficacy, digital literacy, and technology readiness, but it is more narrowly focused on perceived capabilities and abilities related to AI and applying those skills in work-task contexts (Blut & Wang, 2020; Wang et al., 2023). As such, it captures a domain-specific form of perceived readiness that may be particularly relevant in AI-augmented work environments. At the same time, these evolving dynamics involving AI skills and their application for work may heighten social pressures and increase the salience of social comparison in the workplace: as the benefits of AI are often contingent on employees’ ability to use these technologies effectively, differences in AI-related competencies may become increasingly visible among workers (Bankins, Hu, & Yuan, 2024). Such visibility may further encourage employees to evaluate their own capabilities relative to colleagues and work that is AI-enabled (Ma & Li, 2026). Against this backdrop, using three-wave longitudinal data, the present study examines the role of social comparison in shaping experiences of work exhaustion during a period of accelerated AI adoption.

Social comparison is a psychological process through which individuals evaluate their abilities and self-worth in relation to others (Festinger, 1954; Gerber et al., 2018). Social comparison orientation [SCO] (Gibbons & Buunk, 1999) captures individual differences in the tendency to engage in social comparisons with others. Individuals high in SCO are prone to gather information about others in relation to self, and they are more likely to experience negative affectivity. Life satisfaction of individuals high in SCO is more likely to be affected by their immediate environment compared to those lower in SCO (Schneider & Schupp, 2014). In the workplace, employees often use social comparison to assess their performance and skills, which may foster pressure, competition, and even envy (Li et al., 2023; Rinker et al., 2025; Watkins, 2021). This can have negative consequences for job performance and well-being (Buunk & Schaufeli, 2018; Huang & Fan, 2022; Watkins, 2021). Individuals high in SCO may also be more concerned about their job security. Although such social processes are natural, social comparisons may heighten attention to peers who are superior in status or performance, thereby increasing perceptions of inadequacy or threat (Tor & Garcia, 2023).

Social comparison has a central role in social relations and group behavior, but it has received limited attention in population-based studies on occupational health. The growing integration of AI into work provides a novel context in which social comparison unfolds, and its consequences for work well-being are not yet known. Unlike traditional work environments, AI-mediated work restructures existing work practices while increasing digital visibility, algorithmic performance monitoring, and reducing direct social cues (Lazaroiu et al., 2025). These features may fundamentally influence how employees evaluate themselves in relation to others. In such environments, workers may increasingly compare their ability to effectively use AI tools, interpret outputs, and maintain productivity in relation to others. Emerging research on technostress and techno-insecurity suggests that these processes can give rise to techno-comparison, where individuals evaluate their technological competence relative to peers, potentially contributing to strain, threat experiences, and reduced well-being (Li et al., 2025; Nastjuk et al., 2024). As AI tools actively change the nature of many jobs, including those previously unaffected by digitalization, workers increasingly rely on digital systems for planning, decision making, and task execution. In the diffusion of new technologies, adoption typically follows a progression from innovators and early adopters to later adopters and laggards (Dearing & Cox, 2018). Workers who adapt quickly to AI-driven changes may gain advantages in skill development and perceived employability. However, it is also conceivable that early adopters may face heightened expectations and evaluative pressure in AI-augmented work environments. These dynamics suggest that social comparison processes may play a central role in impacting how employees experience AI-related workplace changes and work exhaustion.

Thus, within this context, the integration of AI tools into daily work routines may shift or intensify social comparison among employees by increasing the visibility and evaluability of performance. For example, workers with lower perceived technological competence may compare themselves to colleagues who use AI effectively, signaling aptitude, adaptability, and added value at work. Such comparisons may contribute to feelings of inadequacy and pressure to upskill. In recent research, perception of co-workers AI utilization has been linked to reduced organizational self-esteem and higher anxiety, leading to knowledge-hiding behavior (Xu et al., 2026). Such experiences could strain well-being, particularly when employees’ skills are misaligned with evolving job demands (Cramarenco et al., 2023). At the same time, employees who are more proficient in AI are not necessarily protected from strain (Passalacqua et al., 2025). Although they may benefit from smoother workflows, they may also become more attentive to differences in peer performance and engage in heightened competitive or evaluative social comparisons. Moreover, AI-ready employees may face higher expectations, responsibility, and performance pressure (e.g., “techno-responsibility”), which can transform AI-related competencies from a resource into a demand. More broadly, AI-mediated work environments are likely to have increased digital visibility, transparency of performance, and opportunities for algorithmic benchmarking, thereby amplifying social comparison processes and their consequences for employee well-being.

Amid these developments, little empirical research has examined how social comparison tendencies interact with AI use frequency and AI readiness to influence work exhaustion. Existing studies have rarely incorporated longitudinal data or considered how individual dispositions, such as SCO, may influence vulnerability to strain in technologically advanced work contexts. To address this gap, we use three-wave panel survey data from Finnish employees to investigate whether social comparison tendencies predict subsequent work exhaustion, and whether this association depends on levels of AI use and readiness of use in the workplace. We propose the following research question: Are employees with high social comparison tendencies more likely to experience work exhaustion in work contexts undergoing AI adoption?

**Fig. 1:**
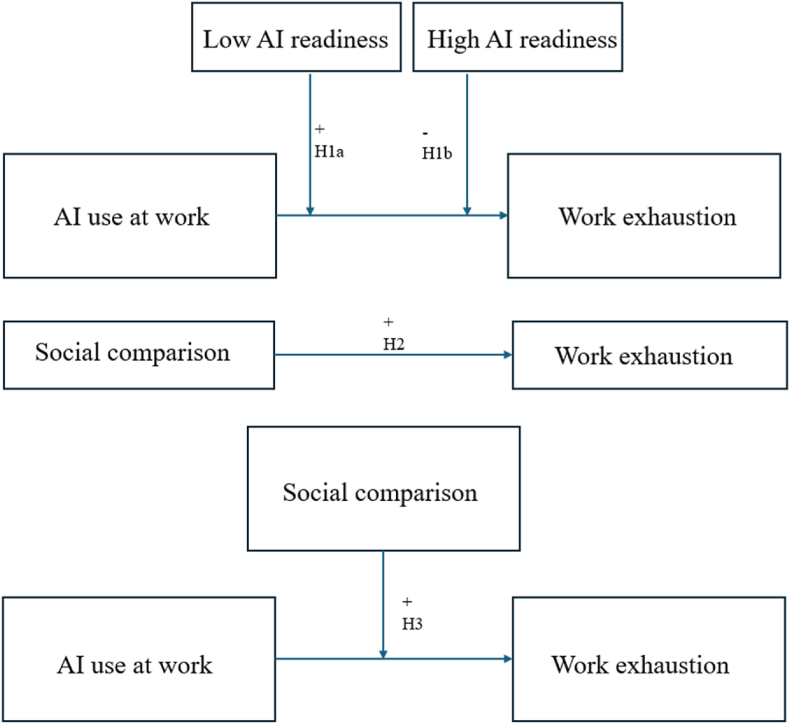
Framework of the relationships between the key variables.

Our analysis draws on literature from occupational health, social comparison theory, and technology adoption models to inform our examination of how AI use frequency and readiness in the workplace are associated with work exhaustion. We propose that AI represents both a technological resource and demand that can either alleviate or intensify strain depending on employees’ readiness to use it. Workers with higher AI readiness may benefit from increased efficiency and autonomy, whereas those with lower readiness may experience greater uncertainty, skills pressure, or perceived obsolescence. The analytical framework assumes that AI use at work is associated with work exhaustion. AI readiness is conceptualized as a personal resource that may condition the association between AI use and exhaustion. In addition, social comparison orientation is expected to be independently associated with work exhaustion and to potentially moderate the relationship between AI use and exhaustion by increasing sensitivity to performance differences and evaluative pressures in the workplace. Sociodemographic and work-related characteristics (age, gender, technological education, supervisory position) were treated as potential confounders, as they may influence both exposure to AI-related work practices and levels of work exhaustion. Fig. 1 presents a conceptual diagram of the assumed relationships.

Given the prior literature covered, we propose the following hypotheses:

H1a Frequent AI use at work is associated with higher work exhaustion among workers with low AI readiness.

H1b Frequent AI use at work is associated with lower work exhaustion among workers with high AI readiness.

H2 Higher social comparison tendency is positively associated with work exhaustion.

H3 Social comparison tendency moderates the relationship between AI use and work exhaustion, such that higher social comparison tendency is associated with stronger positive associations between AI use and exhaustion.

Study hypotheses were preregistered on the Open Science Framework before data collection was finalized (osf.io/s7g46). The original preregistration included a general hypothesis regarding the moderating role of social comparison tendency. In the present manuscript, this hypothesis (H3) is specified more precisely to reflect the direction of the expected interaction and to align with the analytical approach. This clarification does not change the original intention of the preregistered hypothesis.

## 2 Methods

### 2.1 Study design and participants

Data for this study were collected as part of a longitudinal AI Disruption at Work research project examining AI-driven transformations in working life. We used a survey design targeting employees residing in mainland Finland. Participants were recruited via Norstat's online panel, which consists of pre-registered respondents who are part of a closed and invitation-only panel. Sampling quotas were applied to reflect the Finnish working population with respect to age, gender, and region. Eligible participants were adults aged 18–65 who were currently employed. Data were collected biannually across three time points: the first wave (T1) in Fall 2024 (N = 2109), the second wave (T2) in Spring 2025 (n = 1680) and the third wave in Fall 2025 (T3, n = 1479). The follow-up surveys were sent to all T1 respondents and response rates were high: 80·0% at T2 and 70·1% at T3. Upon inspection, the sample closely matched the Finnish working population in key sociodemographic characteristics as reported by Statistics Finland. For example, the mean age of participants (42.6 years) was comparable to the population average (42.2 years), and the gender distribution was nearly identical (50.0% men in both the sample and population). The proportion of respondents with a university degree was also similar (19.4% vs. 18.2%). We found no major deviations due to attrition between timepoints. Attrition analyses comparing participants who remained in the study with those lost to follow-up indicated minor differences in age and baseline exhaustion, with participants lost to follow-up being slightly younger and reporting somewhat higher exhaustion. We observed no differences in baseline AI readiness (P = 0.97).

The data collection procedures were reviewed by the local academic ethics committee, which concluded that the project did not contain any ethical concerns. Informed consent was obtained by notifying the participants that completing the survey indicated their voluntary agreement to participate. Completing the survey took, on average, 15 min. All data were handled anonymously, and the research team received only pseudo-anonymized data from Norstat.

## 3 Measures

Work exhaustion was the dependent variable. It was measured using the Maslach Burnout Inventory Manual, Fourth Edition (Maslach et al., 2018; Maslach & Jackson, 1981), which has been widely validated in research on work-related burnout. We applied the five items assessing emotional exhaustion that can be used across occupational settings. Respondents indicated how often they experienced feelings such as “I feel tired when I get up in the morning and have to face another day on the job” on a scale from 0 (never) to 6 (daily). The scale demonstrated excellent internal consistency across time points in our sample, with McDonald's omega coefficients ranging from 0·92 at T1 to 0·93 at T2 and T3. Exhaustion was treated as a continuous variable in the analyses.

Social comparison was the main independent variable. It was assessed with the six-item version of the Iowa-Netherlands Comparison Orientation Measure [INCOM] (Schneider and Schupp, 2014). The items capture general tendencies to engage in social comparison (e.g., “I always pay a lot of attention to how I do things compared with how others do things”), with responses provided on a scale from 1 (does not describe me at all) to 7 (describes me completely). The scale showed good internal consistency in the sample (T1: ω = 0·85; T2: ω = 0·86; T3: ω = 0·85).

AI readiness was the other main independent variable and was conceptualized as perceived competence, understanding of, and preparedness to engage with AI technologies at work. It was assessed with five items developed by the research group to capture self-reported understanding and knowledge of AI technology, familiarity with AI tools, and perceived ability to apply them at work (e.g., “I know how to use AI tools or products to improve the efficiency of my work”; “I can skillfully use AI tools or products to help me in my daily work”). Responses were given on a scale from 1 (does not describe me at all) to 7 (describes me completely). The items were summed to form a composite scale with higher scores indicating greater perceived AI readiness. The scale demonstrated good internal consistency across all measurement points (T1: ω = 0·82; T2: ω = 0·83; T3: ω = 0·83). We evaluated the factorial structure and longitudinal consistency of the AI readiness measure using confirmatory factor analysis. The configural model showed acceptable fit (CFI = 0·94, TLI = 0·91), whereas the RMSEA (0·10, 90% CI [0·09, 0·10]) and SRMR (0·09) indicated marginal fit. This pattern suggests that the factorial structure of the measure is supported only partially and should therefore be interpreted with some caution. Indicators of convergent validity and internal consistency were satisfactory across all time points, with average variance extracted (AVE) ranging from 0·53 to 0·55 and composite reliability (CR) from 0·83 to 0·85, supporting the use of the measure in the present analyses, but warranting further validation.

Respondents’ use of AI technologies was measured with two items. Frequency of AI use at work was assessed using a single-item measure inquiring how often the respondent used AI in their work with response options ranging from 0 (never) to 5 (several times a day). For the first part of the analyses, this variable was dichotomized to distinguish between frequent (daily) use and less frequent use. Respondents who reported using AI once a day or several times a day were coded as 1 (daily use), whereas all other responses were coded as 0 (less frequent use). Respondents who reported using AI were additionally asked about the types of AI tools they used (e.g., virtual assistants, chatbots, natural language processing models). For the second set of analyses examining social comparison processes, these responses were summed to create a broader indicator reflecting the diversity of AI tool use at work.

Finally, we controlled the models for age, gender (female vs. other), technical educational background, and supervisory position. Age and gender were included as basic sociodemographic factors that may influence both exposure to AI-related work practices and levels of work exhaustion. Technical educational background was assessed at baseline using a binary indicator of whether respondents had completed a degree in a technical field (1 = yes, 0 = no), and was included as a personal resource relevant to AI use and digital competence. Supervisory position was included as a work-related characteristic, as individuals in supervisory roles may differ in their exposure to AI tools, job demands, and levels of work exhaustion.

## 4 Statistical analysis

Descriptive statistics and correlations were obtained for all study variables (Table 1, Table 2). The primary aim of the analyses was to examine contemporaneous within- and between-person associations over time. Therefore, we employed a within–between (hybrid) modeling approach, also known as a Mundlak specification, which allows separation of intra-individual change from stable between-person differences while accounting for repeated measurements. Time was included as a fixed effect to control for overall temporal trends. The analysis fitted linear multilevel mixed-effects regression with restricted maximum likelihood (REML) in R (4·5·2) using lme4 package (1·1·37). Within-person effects (person-mean centered) were decomposed from between-person effects (person means) by including both components separately as fixed effects in the models, allowing estimation of intra-individual and inter-individual associations (Hamaker & Muthén, 2020). Random intercept for individuals was added to account for the repeated measures of the data.

**Table 1:** Descriptive statistics of the study variables.

Continuous variables	Observed range	T1, *M* (*SD*)	T2, *M* (*SD*)	T3, *M* (*SD*)
Work exhaustion	0–30	14·89 (7·98)	14·66 (7·97)	14·54 (8·07)
Social comparison	6–42	21·96 (7·66)	21·46 (7·63)	21·77 (7·40)
AI readiness	5–35	16·90 (5·71)	17·11 (5·56)	17·43 (5·54)
Age	18–65	42·64 (11·95)	44·26 (11·67)	45·48 (11·57)
**Categorical variables**		**T1, *n* (%)**	**T2, *n (%)***	**T3, *n* (%)**
Daily AI use at work		284 (42·26)	297 (51·12)	290 (53·90)
Gender				
Male		1055 (50·02)	883 (52·56)	761 (51·45)
Female		1044 (49·50)	797 (47·44)	718 (48·55)
Other		8 (0·38)	N/A	N/A
Does not want to respond		2 (0·09)	N/A	N/A
Technical degree		807 (28·56)		
Supervisor		170 (18·05)	145 (19·73)	121 (18·97)

*Note*. Descriptive statistics are reported separately for each wave using all available observations (unbalanced panel). Sample sizes vary across waves due to attrition (T1: N = 2109; T2: n = 1680; T3: n = 1479). *AI use at work* is presented as the proportion of respondents reporting daily use of AI tools (coded as once a day or several times a day). *Gender* categories “Other” and “Prefer not to respond” are not shown at later waves due to no respondents in these cells following attrition.

**Table 2:** Pairwise Pearson correlations of the study variables at T1.

	1	2	3	4	5
1. Work exhaustion	1				
2. Social comparison	0·27∗∗∗	1			
3. AI readiness	−0·14∗∗∗	0·06	1		
4. AI use at work	0·03	0·08∗∗	0·35∗∗∗	1	
5. Age	−0·19∗∗∗	−0·38∗∗∗	−0·16∗∗∗	−0·06	1
6. Female	0·15∗∗∗	0·06	−0·21∗∗∗	−0·09	−0·04
7. Technical degree	−0·06	−0·02	0·16∗∗∗	0·12∗∗∗	−0·01
8. Supervisor	−0·06	0·00	0·07∗	−0·00	0·02

*Note*. ∗*p* < 0.05, ∗∗*p* < 0.01, ∗∗∗*p* < 0.001.

Both within and between effects are theoretically meaningful for understanding psychosocial processes in the context of AI-driven work transformation. First, we tested H1a, H1b using work exhaustion as the outcome, including AI readiness (coded as above or below the individual's mean level), frequency of AI use at work, and their interaction. Next, models including the interaction between social comparison and AI use at work were estimated to test H2, H3. Standardized regression coefficients were obtained post-hoc using the parameters package (version 0·28·3). Missing data were handled using listwise deletion at the observation level, such that all available observations with complete data on the variables included in each model were used in the analyses. No imputation of missing values was performed.

## 5 Results

Descriptive statistics across the three measurement waves showed that mean levels of work exhaustion, social comparison tendency, and AI readiness remained relatively stable over time (Table 1). Nevertheless, within-person standard deviations indicated meaningful intra-individual variation in work exhaustion (SDwithin = 3.13), social comparison tendency (SDwithin = 3.07), and AI readiness (SDwithin = 2.27). Across waves, 42–53% of participants who reported using AI at work, used it daily. Table 2 presents pairwise Pearson correlations among the study variables at T1. Social comparison was positively correlated with work exhaustion (*r* = 0·27, P < 0·001), whereas AI readiness was negatively correlated with exhaustion (*r* = −0·14, P < 0·001). AI use at work was positively correlated with social comparison tendency (*r* = 0·08, P = 0·010).

According to the hybrid model testing H1a, H1b, neither AI readiness nor AI use at work showed significant direct within-person associations with work exhaustion. The interaction between AI readiness and AI use at work was also not significant. At the between-person level, capturing stable differences between individuals, AI readiness showed a negative direct association with exhaustion (β = −1.41 [95% CI -2.17 to −0.64], P <0·001). The interaction between AI readiness and frequency of AI use at work was not statistically significant at the between-person level. Among the covariates, individuals coded as female reported higher levels of work exhaustion compared to the reference group (β = 1·00, 95% CI 0·53 to 1·47, P < 0·001). Younger age was also associated with higher exhaustion (β = −1·80, 95% CI -2·27 to −1·33, P < 0·001). Supervisory position and technical educational background were not significantly associated with work exhaustion. These results are presented in Table 3.

**Table 3:** Hybrid multilevel regression analyses predicting work exhaustion (H1a,b).

	β	SE	P	95% CI
Within-person effects				
AI use at work	0·09	0·16	0·547	−0·22, 0·42
AI readiness	−0·04	0·27	0·887	−0·58, 0·50
AI work x AI readiness	0·42	0·64	0·519	−0·85, 1·68
**Between-person effects**				
AI use at work	−0·16	0·53	0·765	−1·19, 0·88
AI readiness	−1·41	0·40	<·001	−2·17, −0·64
AI work x AI readiness	0·69	0·37	0·061	−0·03, 1·43
Female	1·00	0·24	<·001	0·53, 1·47
Age	−1·80	0·24	<·001	−2·27, −1·33
Technical degree	0·08	0·23	0·739	−0·38, 0·53
Supervisor	0·01	0·17	0·965	−0·32, 0·34

Testing Hypothesis 2, social comparison orientation showed a clear and consistent direct association with work exhaustion. Within individuals, increases in social comparison over time were associated with higher exhaustion (β = 0·02, [95% CI 0·01 to 0·04] P = 0·001). Between individuals, those who generally reported higher social comparison orientation also had significantly higher exhaustion levels (β = 0·20, [95% CI 0·15 to 0·25], P < 0·001). For Hypothesis 3, the interaction between social comparison and AI use at work was not statistically significant in either the within-person or between-person components of the model. Female gender (β = 0·11, [95% CI 0·07 to 0·15] P < 0·001) and younger age (β = −0·12, [95% CI -0·16 to −0·08] P < 0·001) were related to higher work exhaustion also in the social comparison model. These results are reported in Table 4.

**Table 4:** Within-between multilevel regression analyses predicting work exhaustion (H2,H3).

Intercept	β	SE	P	95% CI
	15·76	1·25	<0·001	13·32, 18·21
**Within-person effects**				
AI use at work	0·02	0·01	0·053	0·00, 0·03
AI readiness	−0·02	0·01	0·002	−0·04, −0·01
Social comparison	0·02	0·01	0·001	0·01, 0·04
AI work x social comp.	0·01	0·01	0·363	−0·02, 0·04
**Between-person effects**				
AI at work	−0·00	0·07	0·966	−0·13, 0·13
AI readiness	−0·16	0·03	<0·001	−0·21, −0·11
Social comparison	0·20	0·03	<0·001	0·15, 0·25
AI work x social comp.	0·01	0·01	0·439	−0·02, 0·04
Female	0·11	0·02	<0·001	0·07, 0·15
Age	−0·12	0·02	<0·001	−0·16, −0·08
Technical degree	0·01	0·02	0·746	−0·03, 0·05
Supervisor	−0·01	0·01	0·344	−0·04, 0·01

As an exploratory analysis not reported in the main tables, we examined whether associations differed among individuals with higher-than-average levels of social comparison orientation. In this subgroup, the interaction between AI use at work and social comparison was positively associated with work exhaustion at the within-person level (β = 0·05, P = 0·035), but not at the between-person level.

## 6 Discussion

This nation-wide study with 18 months of follow-up examined whether AI readiness, frequency of AI use at work, and social comparison orientation were associated with work exhaustion among Finnish workers. Longitudinal analyses indicated that AI use at work, in itself, was not significantly associated with exhaustion at either the within-person or between-person levels. Higher AI readiness had negative within- and between-person associations with exhaustion, suggesting that perceiving oneself as prepared for AI may protect workers against work exhaustion. However, as we expected that frequent AI use at work would associate with higher or lower work exhaustion based on the level of AI readiness, H1a, H1b were not supported.

The results indicate that frequent AI use at work may not inherently increase job demands or strain. This is also consistent with recent findings reporting no adverse effects of AI use on workers' well-being or mental health (Giuntella et al., 2025). Instead, it may function as a neutral factor at work or potentially a resource, for example, supporting task efficiency or decision-making (Balcerzak et al., 2025), although identifying these mechanisms was beyond the scope of this research per se. In contrast, this study suggests that individuals who perceive themselves as more competent in using AI technologies may be better equipped to cope with new technology-related demands. Within the JDR model, AI readiness can be interpreted as a personal resource that may facilitate adaptation to technological changes and reduce strain. The findings indicate that individual capabilities and perceptions related to AI might be more relevant for understanding work exhaustion than mere frequency of its use. Rather than relating to increased work demands directly, AI use may be experienced differently depending on workers' personal resources. This interpretation highlights the importance of supporting employees’ competencies in tasks where AI is used.

Social comparison orientation was positively associated with work exhaustion at both the within-person and between-person levels, indicating that it was related to both stable individual differences and within-person variation over time. Thus, Hypothesis 2 was supported. These findings are consistent with prior research linking social comparison orientation to adverse outcomes in work contexts (Huang & Fan, 2022). Generally, individuals who are more inclined to engage in social comparison tend to react more strongly to others' abilities and opinions, making their well-being more susceptible to influences from the social environment and they are also more likely to experience negative outcomes (Schneider & Schupp, 2014). These tendencies may increase vulnerability to work exhaustion. For example, individuals high in social comparison orientation might be more prone to overextend themselves as a response to work demands or perhaps respond more strongly to social information about coworkers’ performance.

From the perspective of the job demands-resources model (Demerouti et al., 2001), SCO may function as a dispositional factor that influences how individuals perceive and respond to job demands. Individuals high in social comparison are likely to experience greater evaluative pressure and reduced perceived resources, such as job security, as they habitually assess their standing and labor market value relative to others (Schneider & Schupp, 2014). Although such comparisons can provide self-relevant information, they may also undermine coping capacity and increase susceptibility to strain in changing work environments. We did not observe a statistically significant interaction between SCO and AI use at work in the primary within- or between-person models. Therefore, hypothesis 3 was not supported in the primary analyses. This suggests that, at the population level, the association between social comparison and exhaustion does not systematically depend on the frequency of AI use.

However, exploratory subgroup analyses indicated that among individuals with higher-than-average social comparison tendencies, increases in AI use were associated with higher exhaustion at the within-person level. These findings should be interpreted with caution as they were not part of the preregistered analyses and may reflect patterns specific to the sample. Yet, they are consistent with the possibility that AI-mediated work environments, characterized by increased performance visibility and opportunities for comparison, may intensify strain among those workers who are particularly sensitive to social evaluation. Taken together, these results suggest that SCO is a consistent predictor of work exhaustion but its interaction with AI use seems to be context-dependent and warrants further investigation.

These results highlight the importance of not treating employees as a homogeneous group, as the implications of AI use may differ across individuals. Employees with stronger social comparison tendencies may be particularly sensitive to work demands and performance-related differences, and AI adoption at work may provide an additional context in which these processes are activated. Emerging literature further suggests that workers may not only compare themselves to colleagues, but also to AI systems or AI-assisted coworkers, potentially adding another layer to workplace social comparison processes and experiences of exhaustion (Ma & Li, 2026). Beyond individual differences, the manner in which AI is implemented and used at work may also influence its implications for employee well-being. For example, overreliance on AI or reduced perceived control may exacerbate harmful social comparison processes. Together, these factors underscore the need to attend to situational and organizational conditions that may heighten social comparison at work.

## 7 Limitations and future directions

Key strengths of our study include the longitudinal design and a population-based sample followed across three time points. However, this study has several limitations. The data were drawn from a single national context, Finland, which reflects a Nordic working environment characterized by high levels of technological readiness and may not fully represent variation across occupations, industries, or organizational contexts. All measures were based on self-reported information, which can introduce common method bias. In addition, the single-item frequency measure of AI use at work does not capture qualitative differences in how AI tools are used. For example, it cannot distinguish between routine and more task-specific or critical use. Future research should employ more detailed and multidimensional measures to better understand how different types of AI use relate to work exhaustion and social comparison processes. The items measuring AI readiness were newly created for this study. Although reliability indices and indicators of convergent validity were satisfactory, the CFA indicated only marginal overall model fit. These findings provide partial support for the use of the measure in the present analyses, but further validation is needed to establish its construct validity and to distinguish it from related constructs such as digital literacy or self-efficacy.

The observational design of our study means that residual confounding by unmeasured factors cannot be ruled out. Although the within–between modeling approach helps separate intra- and inter-individual variation, it does not eliminate bias from unobserved variables that may influence both AI-related exposures and work exhaustion. Additionally, the analyses did not incorporate lagged effects or dynamic panel structures, which limits the ability to examine temporal ordering or potential delayed effects of AI use and social comparison on work exhaustion.

Future research should further examine how AI-related workplace changes interact with psychosocial processes over time. In particular, studies are needed to identify organizational and situational factors that impact social comparison dynamics in AI-mediated work. Future cross-national research could further clarify whether the associations between AI readiness, social comparison, and work exhaustion differ across countries with varying levels of technological adoption, workplace digitalization, and labor market structures. Potential moderating effects should also be tested for more comprehensive examinations. Future research should also employ alternative and more detailed longitudinal designs, including lagged or cross-lagged modeling approaches, to better capture temporal dynamics and potential reciprocal relationships between AI use, social comparison processes, and work exhaustion.

## 8 Conclusions

This study examined how AI use at work, perceived AI readiness, and social comparison orientation (SCO) relate to work exhaustion in a longitudinal population-based sample. The findings highlight SCO as the most consistent predictor associated with work exhaustion, both within and between individuals over time. This underscores the importance of psychosocial dispositions in influencing employee well-being in technology-mediated work environments. From a theoretical perspective, the study contributes to existing literature by integrating social comparison theory with research on AI use at work within a longitudinal framework. The findings suggest that social comparison processes may be an important psychosocial factor associated with work exhaustion, even when AI use itself was not directly associated with exhaustion. Within the JDR framework, SCO might function as a dispositional factor that amplifies perceived demands, whereas AI readiness may act as a personal resource. However, conclusions regarding the role of AI readiness should be interpreted with some caution pending further validation of the measure.

Our exploratory analyses indicated that AI use may be associated with higher exhaustion among certain subgroups, however, these findings warrant further investigation. From a practical perspective, the findings suggest that organizations should not focus only on increasing workers’ technical readiness for AI adoption but also consider the psychosocial work environment. Managers can support employee well-being by clearly communicating expectations regarding AI use, ensuring that AI-related responsibilities are aligned with available resources, and avoiding disproportionate workload allocations to those employees who are more AI-ready. In addition, organizations may benefit from interventions that reduce excessive competitive comparison, such as promoting collaborative work practices, fostering transparency in evaluation criteria, and supporting a psychologically safe work climate. These approaches can also contribute to population health by improving well-being at the workforce level.

## CRediT authorship contribution statement

**Iina Savolainen:** Writing – review & editing, Writing – original draft, Visualization, Methodology, Investigation, Funding acquisition, Formal analysis, Data curation, Conceptualization. **Teijo Osma:** Writing – review & editing, Writing – original draft, Methodology, Investigation, Formal analysis. **Roope Grönroos:** Writing – review & editing, Writing – original draft, Visualization, Investigation, Conceptualization. **Moona Heiskari:** Writing – review & editing, Writing – original draft, Investigation, Data curation, Conceptualization. **Atte Oksanen:** Writing – review & editing, Writing – original draft, Visualization, Validation, Supervision, Resources, Project administration, Methodology, Investigation, Funding acquisition, Formal analysis, Data curation, Conceptualization.

## Ethical statement

This study was conducted in accordance with the ethical standards of Tampere University and with the principles of the Declaration of Helsinki. The Ethics Committee of the Tampere Region reviewed the study protocol prior to data collection and concluded that it raised no ethical concerns. All participants were informed about the purpose of the study, and informed consent was obtained from all participants through completion of the survey. Participation was voluntary, and participants were assured of the confidentiality and anonymity of their responses. Contact details for the research team were provided should participants wish to obtain further information about the study.

## Author disclosures

The authors do not have any conflicts of interest to declare.

This work was supported by the Finnish Work Environment Fund (AI Disruption at Work -project, 2024–2026, PI: A. Oksanen, decision number 230330).

## Data Availability

Data will be made available on request.
